# Early diaphragmatic plication after cardiac surgery: a case report in an obese patient

**DOI:** 10.1186/s13019-018-0780-z

**Published:** 2018-09-26

**Authors:** Valérie Lafrenière-Bessi, Frédéric Jacques, Richard Baillot, Jean Bussières, Paola A. Ugalde, Stephan Langevin

**Affiliations:** 1 Institut universitaire de cardiologie et de pneumologie de Québec, Multidisciplinary Department of Cardiology, Service of Cardiac Surgery, Université Laval, Québec, Canada; 2 Institut universitaire de cardiologie et de pneumologie de Québec, Department of Anesthesia, Université Laval, Québec, Canada; 3 Institut universitaire de cardiologie et de pneumologie de Québec, Multidisciplinary Department of Respirology, Service of Thoracic Surgery, Université Laval, Québec, Canada; 4 Institut universitaire de cardiologie et de pneumologie de Québec, Department of Specialized Medecine, Service of Intensive Care Medicine, Université Laval, Québec, Canada

**Keywords:** Diaphragmatic plication, Ventilation weaning, Cardiothoracic surgery

## Abstract

**Background:**

Diaphragmatic plication to help ventilation weaning of an adult obese patient after cardiac surgery is very uncommon. Diaphragm paralysis is usually treated with conservative measures and ventilator support, after which surgical management is considered after months of medical monitoring.

**Case presentation:**

We report the case of a morbidly obese patient to whom ventilation weaning was unsuccessful following coronary artery bypass graft operation with mitral valve replacement. A de novo right hemidiaphragm elevation was seen on the chest X-ray. Diaphragmatic plication was performed promptly to treat severe respiratory insufficiency and generated favorable outcomes.

**Conclusions:**

Early diaphragmatic plication could be considered in the postoperative period of cardiothoracic surgery to facilitate management and ventilation weaning in the context of de novo diaphragm paralysis.

## Background

Diaphragm paralysis due to phrenic nerve injury is a well-recognized complication of open-heart surgery [1]. In adults, diaphragmatic paralysis is most of the time of limited clinical importance and transient. Surgical management for adult ventilation weaning is only considered after months of conservative respiratory support and only when respiratory function is severely compromised [2]. Herein, we report the case of a morbidly obese patient to whom an early diaphragmatic plication was done to treat a severe respiratory insufficiency following myocardial revascularization and mitral valve replacement.

## Case presentation

A 66-year-old morbidly obese man (body mass index of 47 kg/m^2^) was medically treated for hypertension, dyslipidemia and coronary artery disease for years. The patient underwent embolization of an occipital epicranial pseudoaneurysm induced by a previous head injury. Immediately after the intervention, the patient experienced a non-ST elevation myocardial infarction for which a coronary angiogram demonstrated a severe triple-vessel disease. A preoperative echocardiogram also showed a severe mitral insufficiency on a mixed mechanism. The patient underwent an uneventful coronary artery bypass graft operation with left internal mammary graft and a trans-septal bioprosthetic mitral valve replacement.

**Fig. 1:**
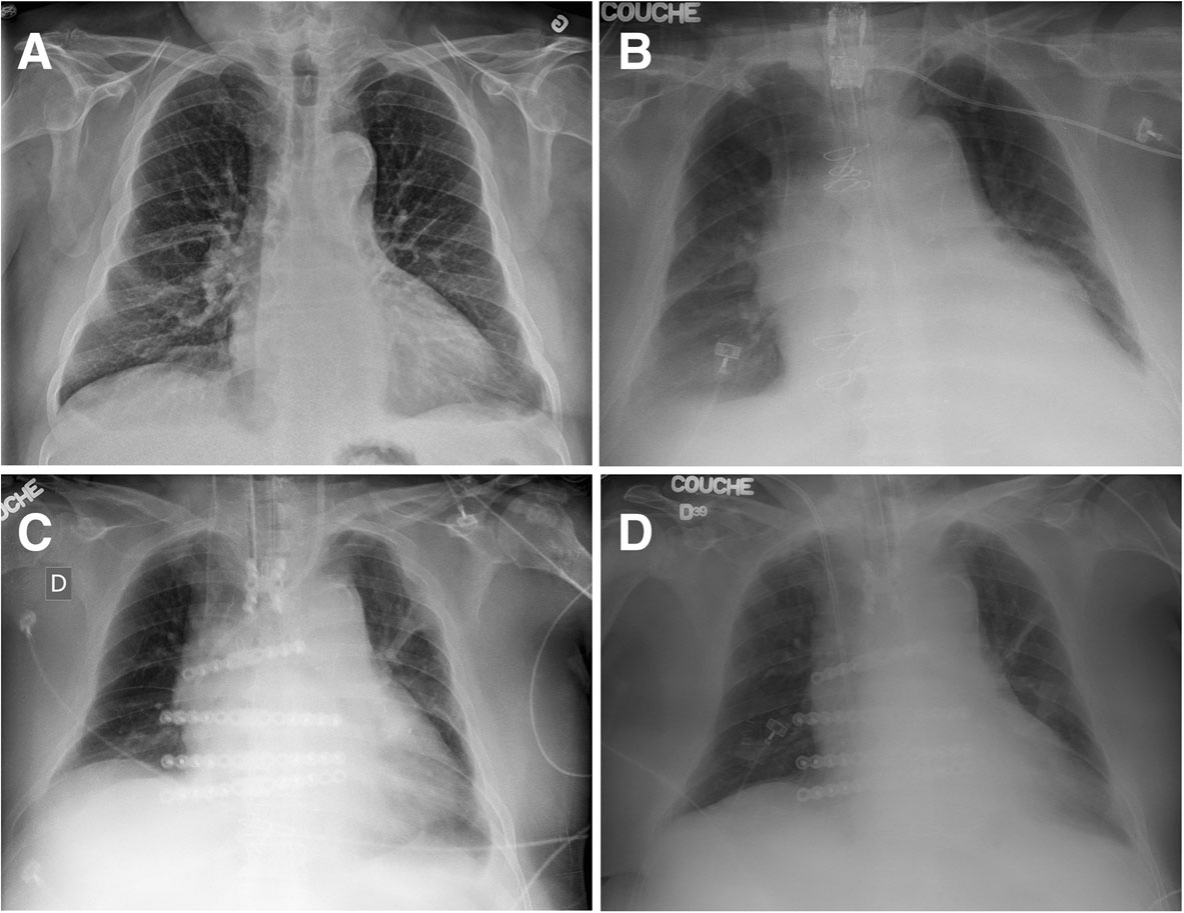
**a** Preoperative chest X-ray. **b** Postoperative chest X-ray immediately after myocardial revascularization and mitral valve replacement. **c** Postoperative chest X-ray immediately after the chest wall reconstruction with plates and screws. **d** Postoperative chest X-ray immediately after diaphragm plication

In the days following surgery, the patient developed severe delirium. On postoperative day 9, a sterile sternal dehiscence was documented for which a sternal reconstruction was done with titanium plates and partial bilateral pectoralis myocutaneous flaps (Titanium Sternal Fixation System, Johnson and Johnson®, Markham, ON). In the immediate postoperative period, the patient had atrial fibrillation witch required direct current cardioversion. De novo right hemidiaphragm elevation was seen on the chest X-ray with hemidiaphragmatic paralysis suspicion (Fig. 1). The patient responded well to the clinical management and was extubated on postoperative day 19. Respiratory insufficiency was treated with non-invasive ventilation that was not well tolerated by the patient, which forced reintubation. A right hemidiaphragm elevation was present on the chest X-ray (Fig. 1c) and a transthoracic ultrasonography confirmed the diagnosis of diaphragmatic paralysis.

After three and a half weeks of conservative management, a decision was made to perform a right thoracoscopic hemidiaphragm plication following informed consent from the family. Plication was performed using resolvable sutures to lower the diaphragm and reinforced sutures were passed through the chest wall and tied at the skin level. The chest X-ray showed a lowered hemidiaphragm immediately after plication (Fig. 1d). The patient was kept intubated for another 5 days, to minimize stress on the sutures. The dynamic compliance increased progressively from 19 to 22 ml/cm measured at the preoperative period to 40–50 ml/cm postoperatively during pressure support ventilation. Subsequently, sedation and mechanical ventilation were discontinued and the patient was successfully extubated. During this period of time, the patient weight, volume status, delirium status, hemodynamic and hepatorenal values were all stable. Chest X-ray on the following days demonstrated better lung expansion on the right chest. The patient recovered and was discharged without any further respiratory distress and complication.

Five months following diaphragmatic plication, pulmonary function testing revealed a forced expiratory volume in the first second of 1.40 L, a forced vital capacity of 1.81 L, a total lung capacity of 3.53 L and a Tiffeneau index of 77%. A follow-up echocardiogram demonstrated a well-functioning mitral bioprosthesis and a normal left ventricular function. Three years later, similar pulmonary function testing was documented and the patient remain in a clinical functional class I (NYHA).

## Discussion and conclusions

Diaphragmatic paralysis is a well-known complication of cardiac surgery. Respiratory function usually recovers over the course of several weeks; therefore, common practice is ventilator support until phrenic nerve recovery [2]. This conservative strategy is used to prevent patient exposure to surgical risks. When respiratory functional recovery is not obtained after several months, diaphragmatic plication may be performed to improve symptoms. Mechanical ventilation weaning and recovery can be especially difficult in some patients and diaphragmatic plication could be considered earlier [1]. In special circumstances such as children with univentricular physiology, the risks of performing a thoracic procedure are way less than leaving a young patient, with higher than normal pulmonary resistance, intubated for several weeks [3].

In the patient presented herein, the “wait and see” strategy did not seem appropriate. Leaving this morbidly obese patient on continuous mechanical ventilation could have led to more severe respiratory complications. Alternative airway management such as a tracheostomy could have lowered the respiratory work of breathing in this patient but would have been associated to an elevated risk of mediastinal infection, deep sternal wound infection and chondritis due to previous sternal plating. It was decided to tailor the approach to the clinical context rather than adhering to the conservative approach. The ease of performing early extubation after plication in this patient seems to support the strategy used herein. The reason as to what caused a right diaphragmatic paralysis in this case is unknown. The intrathoracic surgery was remote from the development of the diaphragmatic paralysis. This surgery did not involve the harvest of the right internal thoracic artery or ice sludge, two well recognized causes of phrenic nerve damage [4, 5]. There was no new central venous line placement at the time of the sternal reconstruction. Interestingly, the patient received direct current cardioversion for atrial fibrillation following sternal reconstruction surgery. Although rare, unilateral diaphragmatic paresis has been reported as a complication of direct current cardioversion while the exact mechanism remains unclear [6]. In addition, no functional neurological exam was performed for this patient in order to evaluate phrenic nerve response, nor the sniff test could be performed due to the clinical state. Making a direct link between diaphragmatic plication for patients with respiratory insufficiency following cardiac surgery and ventilation weaning is hard to pin point. However, clinically, diaphragm plication was an undeniable turning point.

In this obese patient, the restrictive physiology and diaphragm paralysis were associated with decreased respiratory compliance resulting in hypoxia and delirium. The upward hemi-diaphragm movement was likely higher than in non-obese patients leaving less volume available for the right lung to fill. The implication of the right lung, anatomically larger than the left and the reduction of chest wall compliance due to the recent sternal reconstruction were negative influences on respiratory physiology. Following this reasoning, diaphragmatic plication in the early postoperative period after cardiac surgery was decided for this patient’s clinical context. Early diaphragmatic plication in select patients could be considered to facilitate the postoperative management and ventilation weaning in the context of de novo diaphragm paralysis.
